# Towards a World Wide Web powered by generative AI

**DOI:** 10.1038/s41598-024-77301-0

**Published:** 2025-02-28

**Authors:** Nouar AlDahoul, Joseph Hong, Matteo Varvello, Yasir Zaki

**Affiliations:** 1 Computer Science, New York University Abu Dhabi, Abu Dhabi, United Arab Emirates; 2 Nokia Bell Labs, New Jersey, USA

**Keywords:** Computer science, Information technology

## Abstract

Generative Artificial Intelligence (AI) is a cutting-edge technology capable of producing text, images, and various media content leveraging generative models and user prompts. Between 2022 and 2023, generative AI surged in popularity with a plethora of applications spanning from AI-powered movies to chatbots. This paper investigates the potential of generative AI within the realm of the World Wide Web, specifically focusing on image generation. Web developers already harness generative AI to help craft text and images, while Web browsers might use it in the future to locally generate images for tasks such as repairing broken webpages, conserving bandwidth, and enhancing privacy. To explore this research area, this paper developed *WebDiffusion*, a tool that allows to simulate a Web powered by stable diffusion, a popular text-to-image model, from both a client and server perspective. Such a tool is the first of its kind, paving the way towards a futuristic world wide web where web images can be created using generative AI. WebDiffusion further supports crowdsourcing of user opinions, which is used to evaluate the quality and accuracy of 409 AI-generated images sourced from 60 webpages. Our findings suggest that generative AI is already capable of producing pertinent and high-quality Web images, even without requiring Web designers to manually input prompts, just by leveraging contextual information available within the webpages. However, direct in-browser image generation remains a challenge, as only highly powerful GPUs, such as the A40 and A100, can (partially) compete with classic image downloads. Nevertheless, this approach could be valuable for a subset of the images, for example, when fixing broken webpages or handling highly private content.

## Introduction

In the last year, generative Artificial Intelligence (AI) has emerged as a revolutionary technology capable of generating diverse forms of content, including text^1,2^, images^3–7^, and multimedia^8–10^. This surge in interest has ignited a wave of innovative applications across various domains, from the entertainment industry with AI-powered movies^11,12^ to the realms of medicine^13,14^. This paper delves into the fascinating role of generative AI within the context of the World Wide Web.

Web developers are turning to generative AI to automate the creation of images and multimedia assets, or speedup webpages (According to HubSpot Blogs research, 93% of web designers have used an AI tool to assist with a web design-related task in the summer of 2023^15^). Similarly, browser vendors are exploring the potential of generative AI running inside the browser, e.g., Summarizer by Brave^16^ and MAX by Arc^17^. In the future, Web media, such as images, could be locally generated by the browser, offering solutions to rectify broken web pages, optimize bandwidth usage, and enhance privacy protection.

To explore this intriguing intersection between generative AI and the Web, WebDiffusion is introduced, which is a tool that allows to emulate a Web leveraging stable diffusion for image generation, both from a client and a server perspective. This paper focuses on image generation for two reasons. First, images are a vital component of the Web; for example, the median webpage today is comprised of 900 KB of images (or about 44% of its total size)^18^. Second, image generation has no impact on Webcompat^19–21^, differently from AI-based generation of HTML, JavaScript, etc. Still, this is an interesting research question that is left open for future work.

Given a target webpage, WebDiffusion crawls its content, produces AI-based images, and performs experiments with unmodified browsers. Last but not least, WebDiffusion integrates with Prolific^22^, a popular crowdsourcing platform, to gather worldwide eyeballs reporting on the quality of Web images and webpages produced by generative AI. This component of WebDiffusion is currently live at *https://webdiffusion.ai/*. WebDiffusion is used to investigate several research questions.

*Can Web images be automatically generated?* The top 200 webpages from Tranco’s top one million were collected, which contain at least an image, load correctly, and are written in English. From these pages, 1,870 images were collected, for which WebDiffusion generated annotations (prompts) to be used for image generation. Only the client-side information were considered, *i.e.,* alt-text and the content of the webpage, to emulate a generative AI running in the browser, and server-side information, *i.e.,* with the addition of the actual image description and details as an approximation of the Web designer’s intent. With a user study involving 1000 participants, the results show that the client-based approach produces relevant annotations 80% of the time, while this fraction grows to 90% when considering a server-based approach.

*What is the quality of AI-generated webpages?* From the above dataset, 60 webpages were randomly selected (accounting for 409 unique images) and an image (and webpage) was generated for each using stable diffusion. To do so, WebDiffusion rely on the annotations discussed above, thus producing both client-based and server-based versions of each image/webpage. Then, about 940 people (10 per image/webpage, on average) were crowdsourced to score the quality of the AI-generated content. The results show that 70–95% of the images are scored as either “fair” or higher (“good”, “very good”, or “excellent”), and 95% of webpages are scored as “good” or higher. The server-based approach achieves the highest quality, thanks to the additional contextual information available. Furthermore, the paper further investigates which image features, e.g., landscape versus celebrity, have the largest impact on high and low scores. The results show that images including text achieve the lowest scores, followed by images with faces.

*Can image generation run in the client?* By loading multiple webpages under several network conditions, our experiments show that, as of today, only powerful GPUs (e.g., Nvidia Tesla A40 and A100) can compete with actual image download, e.g., not dramatically slowdown a webpage load. Further, this is only true for SpeedIndex, a popular web performance metric relating to only the visible portion of the screen (i.e., what is known as above the fold). This paper analysis found that there is potentially a 2.5-5 second improvement for SpeedIndex leveraging image generation. Moving to PageLoadTime, which instead requires the whole page to be downloaded, then even powerful GPUs would add tens of seconds. In conclusion, such an approach is currently viable for applications operating on a handful of images, with two popular examples being fully privacy-preserving advertising and webpage repair, as discussed in the Discussion Section.

## Background and related work

### Generative AI research

Generative AI has recently received a lot of attention within the research community. There have been multiple avenues and applications of generative AI in various domains. For example, Ibrahim et. al.^23,2^ assessed the potential of generative AI on university level education, where they compared the performance of ChatGPT against university-level students across a variety of courses. Stribling et. al.^24^, on the other hand, also assessed ChatGPT’s performance but on graduate-level examinations in the biomedical sciences. Both works show that ChatGPT exceeded students’ performance in multiple courses. Generative AI has also been used in other fields, such as medicine. For example, Pinaya et. al.^25^ integrated generative AI capabilities in medical imaging tasks such as image reconstruction and enhancement, demonstrating the transformative potential of these technologies in radiology and other imaging-related fields. Ghebrehiwet et. al.^26^ examined the integration of generative AI models in personalized medicine, highlighting their potential to revolutionize patient-centric healthcare by enhancing diagnosis and treatment personalization. In art, the work of Zhou and Lee^27^ explored how text-to-image generative AI systems like Midjourney, Stable Diffusion, and DALL-E impact artistic creativity and production. Their findings indicated that such systems can enhance human creativity by enabling artists to explore novel ideas more effectively, leading to increased productivity and more favorable evaluations from peers. Finally, Aldahoul^28^ studied the extent to which text-to-image models, such as Stable Diffusion, exhibit racial and gender stereotypes. They highlighted the potential and danger of such models and how they can impact the public’s perception. Specifically, they showed that being presented with inclusive AI-generated faces reduces people’s racial and gender biases, while being presented with non-inclusive ones increases such biases.

### Text-to-image generation

A variety of text-to-image generation models have been released over the last few years. These models face the so-called *generative learning trilemma*^29^, *i.e.,* they can only satisfy two out of three requirements that real-world applications of generative models should possess: high-quality outputs, output sample diversity, and computationally fast and inexpensive sampling. Image generation usually requires high-quality outputs, so general adversarial network (GAN) or diffusion models are preferred.

Thanks to recent developments, diffusion models are currently outperforming GAN models^30^. These gradually add Gaussian noise to the input, and afterwards perform a reverse denoising process, which reverses this process and outputs an image^29^. GLIDE and DALL-E 2^6,7^ both use classifier-free diffusion models as a part of their image generation process. These diffusion models produce very realistic outputs and have large mode coverage, ideal for image generation, but tend to be very slow due to the number of iterations required throughout the denoising processes. This is the issue that the latent diffusion model^4^, the foundation of *stable diffusion*^31^, addresses. By transforming the diffusion model to be compatible with a compressed image representation in a latent space, latent diffusion allows the retention of high-quality outputs on a wide variety of image-generation tasks quickly and in a computationally efficient manner^31^. Fulfilling the three aforementioned requirements, latent diffusion has potential for real-world applications, which is our motivation for selecting stable diffusion as the image generation model for this paper.

### On device image generation

Zhao et al.^32^ have recently proposed “MobileDiffusion”, a rapid text-to-image generation on mobile devices. This work explores how to efficiently run text-to-image generation models for mobile devices, enabling the generation of high-quality images directly on users’ devices. This can be particularly useful for web applications that require quick and responsive image generation without relying on server-side processing. Similarly, Castells et al.^33^ proposed “EdgeFusion”, focusing on deploying text-to-image generation models on edge devices using mixed-precision quantization. The research demonstrates significant improvements in performance and efficiency, making advanced generative models more accessible for on-device applications.

### Industry solutions for generative AI

Google has been actively integrating generative AI into various platforms and tools to enhance web experiences. Their efforts include tools for developers like Google AI Studio and Project IDX^34^, which facilitate the integration of generative AI capabilities into web and mobile applications. These tools enable features such as AI-powered content generation and coding assistance, which can significantly enhance web development. Meta, on the other hand, has introduced its own text-to-image generation tool, “Imagine”^35^. Imagine, which is currently being integrated into platforms like Instagram and Facebook, demonstrates how generative AI can be used to create and enhance web content dynamically, providing users with the ability to generate and share AI-generated images directly within these web environments. Finally, Apple has recently introduced “Apple Intelligence”^36^, a system that integrates generative models deeply into iOS, iPadOS, and macOS, allowing for personalized and contextually aware AI features. For instance, it enables natural language processing to search for specific photos and videos and to generate custom memory movies from user descriptions. This helps create more dynamic and interactive web experiences by generating content on-the-fly based on user inputs.

### Image generation for the web

To the best of our knowledge, no previous work has investigated the role of generative AI for Web images consumption. The closest work to this topic is^37^, where the authors propose to replace Web images with *similar* content, e.g., a similar picture of the Golden Gate bridge taken from a different angle. Their rationale is to “race” similar Web images – identified via reverse image searches – to speedup webpage loads. The similarity with our work stands in the idea that Web images are not always “strict”, aka users might be willing to accept similar images to the originals if this comes with some benefits, such as page speedups in^37^. The key difference to this work is that this paper broadly explores the field of generative AI when applied to Web images, considering both a client and server standpoint.

**Fig. 1:**
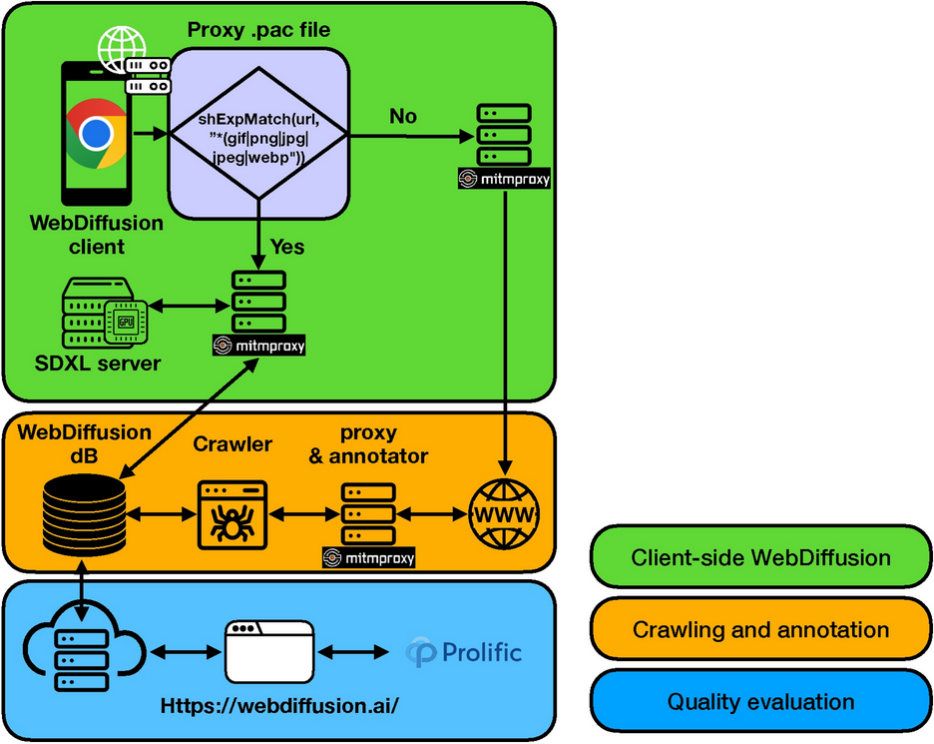
A visualization of WebDiffusion, our tool to evaluate the applicability of text-to-image generation for the Web.

## Methods

This section presents WebDiffusion, a tool that allows the exploration and emulation of a web, leveraging stable diffusion for image generation. WebDiffusion aims at evaluating both a *server* and a *client* mode. The server mode refers to a scenario where Web designers rely on stable diffusion for image generation. The client mode refers to a scenario where image generation is fully run in the browser, e.g., to generate privacy-preserving advertisements or fix broken URLs.

At high level, WebDiffusion consists of the four components visualized in Fig. 1. First, a *crawler* that makes local copies of actual webpages while annotating embedded images with textual prompts using two techniques discussed next. Second, a *proxy* system to replay webpages while replacing images with textual prompts to feed to a *stable diffusion server*, allowing the emulation of an AI-powered Web without requiring an in-browser implementation. Finally, a *Web interface* which collects human feedback on the accuracy of AI-generated images and webpages. Each component of WebDiffusion is described in the remainder of this section.

**Fig. 2:**
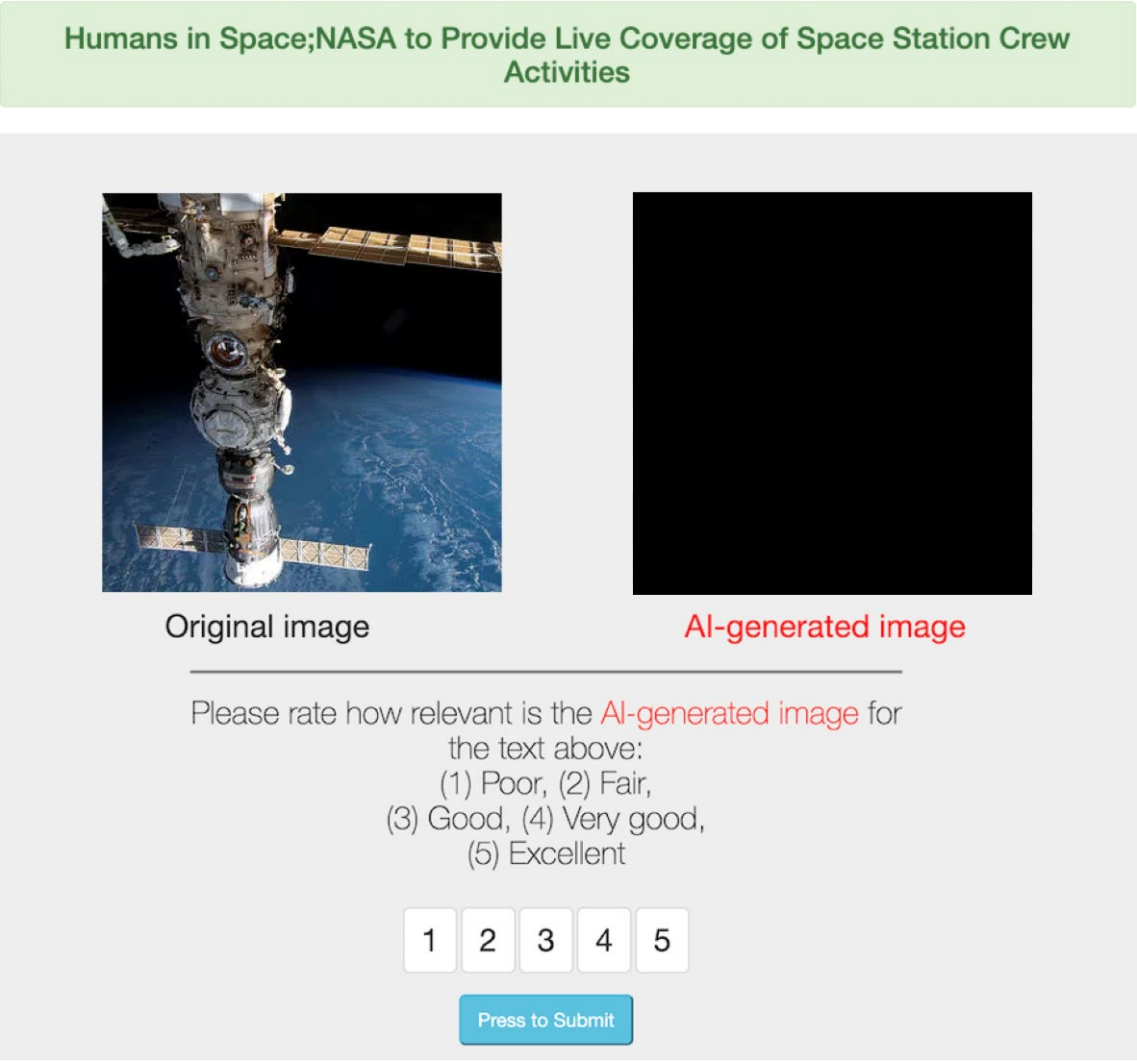
WebDiffusion's GUI. The top of the figure shows the text originated using the client-based approach, *i.e.,* eventual *alt text* and nested divs information. The center of the figure shows both the original and the AI-generated images. The bottom of the figure allows study participants to score the quality of this AI-generated image, between 1 (poor) and 5 (excellent). No exemplary image generated by AI is shown due to the publisher's copyright restrictions. However, the AI-generated image can be found at https://comnetsad.github.io/webdiffusion.html.

### Crawler

The first task of the WebDiffusion's crawler is to generate local copies of webpages. These webpages can be analyzed to investigate the feasibility of image annotation techniques discussed below, as well as “replayed” to benchmark the performance of AI-generated webpages. The crawler consists of a Selenium^38^ application which, for a given webpage, records headers and data returned for each HTTP(S) request along with its duration. Crawled webpages are stored in a database so that they can then be *replayed* via the proxy.

Next, the crawler runs the following annotation schemes to obtain textual prompts for each image embedded in a given webpage. Each textual prompt is also stored in the database, along with the technique used for its derivation.

#### Client-based

This technique aims at being deployed today without any server-side support, meaning that it works solely relying on *contextual* information, e.g., alt text sometimes provided along with an image (about 50% of the time^39^). The trade-off is a potential lack of accuracy as the browser is forced to “guess” the content of an image without having access to it.

This mechanism works as follows: For each image tag in a page’s HTML that has either a src or background-image: url attribute in its CSS styling, WebDiffusion looks for its alt text (or alt tag), whose goal is to provide a description of an image in case the actual image is missing. In addition, it also iterate through the different div elements associated with the image looking for potential text within $<p>$, $<h1>$, $<h2>$, $<h3>$ and $<h4>$ tag elements, and then iterate through their children nodes to extract the text associated with them. This is often where the title and the description of an image are stored. All the text associated with these different elements, is then accumulated into forming the client-based textual prompt to be used to generate the image. It also recursively traverses the parent div nodes of the image div to extract all the text related to the image; it stops when it discovers the presence of another image div and reverts back to the previous node.

#### Server-based

This technique implies some server-side support, e.g., from web developers, to annotate images. It extends the client-based approach with image annotation obtained via Microsoft’s GenerativeImage2Text transformer^40,41^, which derives a textual prompt related to an input image. Note that leveraging GenerativeImage2Text allows us to build an approximation that can be evaluated today without support from web developers.

### Proxy system

In theory, stable diffusion can be integrated into any open-source Web browser. In practice, this is a time-consuming effort, which can anyway lead to suboptimal performance as browsers are very complex software. Instead, WebDiffusion offloads image generation to an external machine (a stable diffusion server) and leverages a proxy system to intercept and operate on Web traffic (see Fig. 1). This setup allows WebDiffusion to control how images should be served, either in a classic way or generated by the stable diffusion server.

As shown in Fig. 1, legacy clients – mobile or desktop running any browser – are instructed to forward all their HTTP(S) traffic via mitmproxy^42^. This requires installing mitmproxy’s root CA (Certificate Authority) to handle HTTPS traffic. Note that this step is only required for testing purposes, as a browser implementation would not require these steps. All traffic is assumed to be HTTP/2^43^.

Our experimental proxy system consists of two mitmproxy proxies: one handling only requests for images, and one serving all the other requests. These two proxies are set in the testing client using a simple Proxy Auto-Configuration (PAC) file^44^ with a regular expression matching requests for images towards one proxy and vice versa (note that this also requires setting the browser’s PacHttpsUrlStrippingEnabled policy to false so as to enable full URL visibility in the PAC file with HTTPS). This is necessary to allow the flexibility to emulate network connectivity (realized via Linux tc^45^) at the link between legacy clients and the proxy not handling image requests. In fact, the network characteristics of this link should not affect the images generated by the stable diffusion server since a client-side implementation is being emulated. This is ensured as long as a fast connection (1 Gbps) is available between the client, image proxy, and stable diffusion server. For each request received, our proxy system performs a database lookup. For non-images, this lookup returns the content to be served. For images, the database lookup might return some textual prompt for image generation. This depends on the experiment running, e.g., some prompts might be missing when testing the client-based approach (see the Stable Diffusion Server section). In the presence of a textual prompt, the proxy contacts the stable diffusion server, which generates the image. The stable diffusion server can be configured with different hardware to emulate different client capabilities. When the image is generated, it is returned to the proxy, which forwards it back to the client.

### Stable diffusion server

Given the prompts derived using any of the above techniques, the image generation is automated, relying on stable diffusion. Stable diffusion is chosen for the reasons discussed in the Background and Related Work section and because of its open source nature. The stable diffusion (SDXL) implementation of huggingface.co was deployed using their stabilityai/stable-diffusion-xl-base-1.0 model^46,47^. It is a latent diffusion model with a three-times larger UNet backbone for text-to-image synthesis that shows remarkable performance compared to previous versions of stable diffusion. SDXL uses a second text encoder, OpenCLIP ViT-bigG/14, and multiple novel conditioning schemes^47^. To create prompts from images, as needed by the server-based technique explained earlier, the large-sized GenerativeImage2Text transformer from microsoft/git-large-coco^40,41^ which was fine-tuned on COCO was utilized to generate an image caption to be used as a text prompt. The architecture of the GenerativeImage2Text transformer consists of one image encoder and one text decoder under a single language modeling task^40^.

The *quality* of images produced by stable diffusion increases with the number of inference steps, which in turn requires a longer generation time. The number of inference steps relates to how many denoising steps the model will use to improve the quality of the generated images. Generally, 50 denoising steps are sufficient to generate high-quality images. While experimenting with stable diffusion in the context of the Web, it was observed that some textual prompts benefit more than others from more iterations, e.g., human faces versus a landscape. Thus, this paper settled for a constant number of iterations (20) for fairness between images from different webpages. This number was chosen as a good compromise between quality and speed (about one second of image generation time on our machine). From the different Web images that were generated, it was observed that the above number of inferences is enough for generating a large portion of the Web images, where any image that does not contain text or faces had a good quality (for example, food, landscape, objects, animals, etc.). However, an interesting direction for future work is to explore some dynamic, stable diffusion settings based on the content of a textual prompt and potentially additional webpage information. Apart from the number of inferences, all other stable diffusion parameters were kept to the default values: guidance scale (5) and width and height (1024x1024 pixels). The seed was randomly generated to have different images generated each time.

Our stable diffusion server currently runs on a Linux server equipped with Nvidia Tesla A40 GPU^48^ with a 48 GB GDDR6 with error-correcting code (ECC), 300 W maximum power consumption, and a 696 GB/s GPU memory bandwidth. The server specifications are: Intel@Xeon(R) Bronze 3204 CPU@1.90GHz x 12, with 16 GiB RAM, and running Ubuntu 20.04.5 LTS.

### Web interface

Last but not least, WebDiffusion offers a Web interface at *https://webdiffusion.ai/*. At this page, users can explore Web images or full webpages automatically generated using the above annotation schemes (examples can be found at https://comnetsad.github.io/webdiffusion.html), while providing some feedback on the quality of the generated content. The Web interface accepts a parameter (?type=[images, webpages]) to control whether to show images or full webpages and which annotation scheme to use – default is server-based, while client-based is activated by adding the suffix _client.

Figure 2 shows a visualization of WebDiffusion's Web interface when instrumented to show an image comparison. At the top of the page, some textual prompts are offered to describe the images. This text was obtained using the techniques described above for image annotation. Next, two images are shown. One image is an original Web image pulled from a given website; the other image is originated via stable diffusion using the above textual prompt. Original images are displayed on the left, whereas the AI-powered images are displayed on the right. Both images are clearly labeled. At the bottom of the page, the user is asked to rank the quality of AI-generated images between 1 (poor) and 5 (excellent). WebDiffusion's Web GUI was integrated with Prolific^22^, a popular crowdsourcing website that allows us to quickly scale user studies. A standard 5-point Likert scale was used with a theme focusing on evaluating quality. The theme uses the following labels (also known as “Likert Scale Response Anchors”): (1) poor, (2) fair, (3) good, (4) very good, and (5) excellent. Notice that the numerical values associated with each label were also provided so as to give the participants a sense of the distance between the labels and to help with processing the data qualitatively during our analysis phase.

**Fig. 3:**
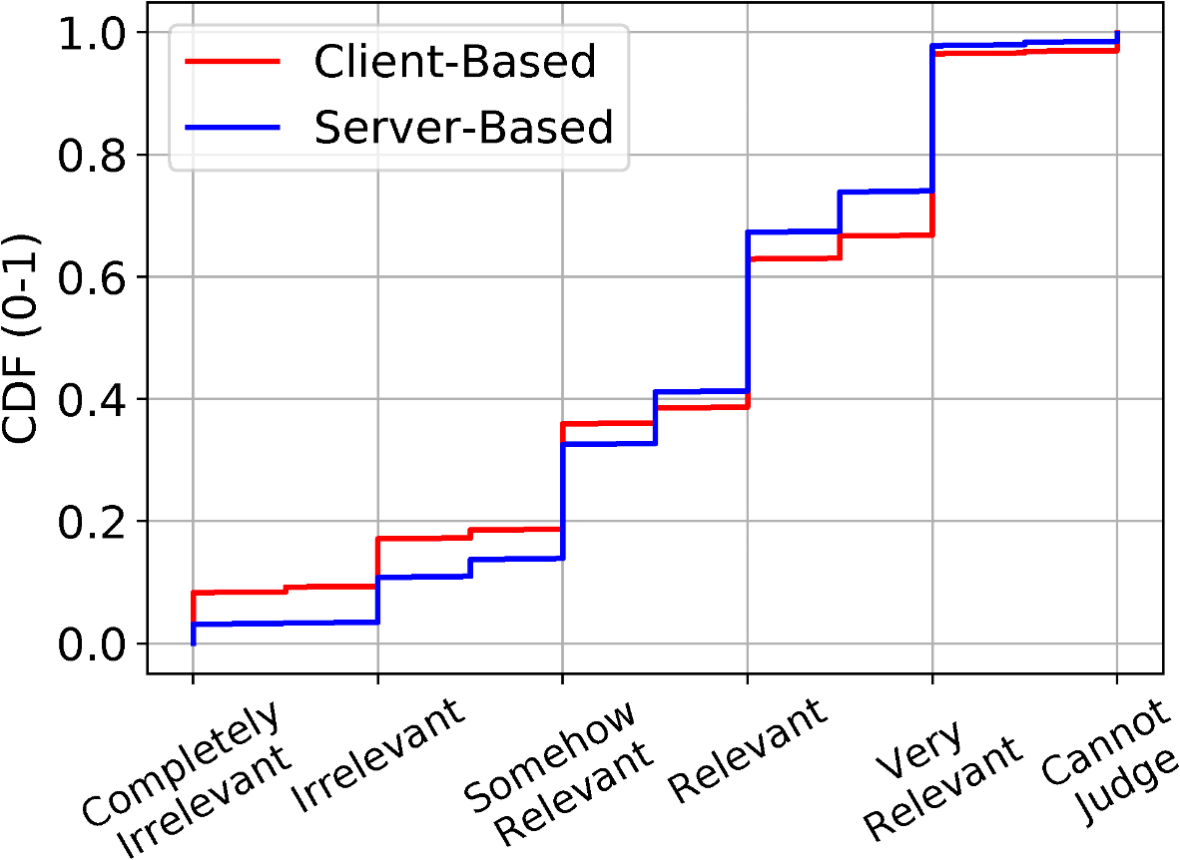
CDF of how relevant client-based and served-based annotations are with respect to original images extracted from webpages (1,870 images from 200 webpages evaluated by 1,000 people each evaluating 10 random images).

## Results on the feasibility, quality, and performance of AI-generated webpages

This section evaluates the applicability of generative AI for the Web, specifically focusing on stable diffusion for image generation^4,5,7^. Our evaluation revolves around three main parts. First, 200 webpages were crawled to quantify the *feasibility* of the techniques discussed in the previous section for image annotation, which is paramount for the generation of the prompts used for image generation. This work considered both client-based annotations, *i.e.,* a best effort to locate webpage text that should refer to an image, and server-based annotations, where an access to the original image is assumed as an approximation of a Web designer’s intent. Next, the *quality* of AI-generated webpages is evaluated, or webpages where images are automatically generated via stable diffusion^4^. This is done by performing several user studies involving about 900 participants provided by Prolific^22^. Finally, the *performance* of AI-generated webpages is benchmarked using the following web performance metrics^49^: SpeedIndex, Page Load Time, and bandwidth usage. WebDiffusion was used to perform all the experiments discussed in this section, leveraging WebPageTest^50^ and Chrome to automate the webpage loads and collect the browser telemetry data.

### Can web images be automatically generated?

The top 500 webpages from Tranco’s top one million list were crawled using Selenium. About 25% of these webpages fail to load; for the remainder 75% of webpages, the crawler achieves a 70% success rate (260 webpages); failures are due to webpage formatting, e.g., lack of img tags with clear src or background-image attributes. Next, webpages hosting inappropriate or non-English content are filtered, given that stable diffusion currently operates only with English prompts. This results in 200 webpages with a total of 1,870 images for which some client-based annotation is produced. Finally, the GenerativeImage2Text transformer is used to derive the extra annotation needed by the server-based scheme. This allowed us to generate a textual description of the original images.

Client-based annotation is a best-effort attempt; as such, potential mistakes are possible. The output of the GenerativeImage2Text transformer is instead highly accurate – given their usage of the BLIP image-captioning technique, which is shown to outperform all state-of-the-art techniques^51^ – but generic. For example, an image of the Golden Gate Bridge might generate a label such as “a bridge with some clouds in the background”; conversely, the text in the webpage might describe more precisely which kind of bridge and its scenery.

The correctness of the annotations is evaluated using Natural Language Processing (NLP) of sentence similarity. The bert-base-nli-mean-tokens^52^ model is used to generate text embeddings for both annotation schemes, each containing 768 values; which is then followed by computing the cosine similarity^53^ between both texts’ embeddings. The results show that both the median and average similarities are about 50% (with 75th percentile at 60%, and 25th percentile at 40%). This result is encouraging since it suggests some intersection between the two annotation schemes. Of course, a perfect intersection is unlikely given that the output of the GenerativeImage2Text transformer is highly generic. However, systematic misbehavior of the client-based annotation scheme would result in much lower cosine similarity values.

To further comment on the quality of automated web image annotations, a user study is performed. WebDiffusion's Web interface is extended (activated with parameter ?type=[scale, scale_prompt], see the Web Interface section) to show a single (original) image along some descriptive text and ask for how *relevant* such text is to the image. Scores are in the range of “completely irrelevant” to “very relevant”. The study participants were also allowed to respond “cannot judge”, in cases when participants were not knowledgeable of the image, e.g., when the text refers to a celebrity they do not know.

**Table 1:** User study summary. *Server* is short for server-based, and *client* is short for client-based.

	Images server	Imagess client	Webpages srver	Webpages client
Size	409	409	60	60
Participants	410	413	60	60
Responses	4110	4160	600	600
Age Range	19–63	19–63	20–40	18–52
Gender (M/F)	239/171	197/216	20/10	20/10
# Countries	29	26	9	10

Figure 3 shows the Cumulative Distribution Function (CDF) of the median score (10,000 scores from 1,000 people) for the 1,870 images that were collected from 200 websites, distinguishing between the client-based and server-based annotations shown on top of each image. The figure shows a success rate – score higher than “irrelevant” – of 80% for the client-side approach, and close to 90% for the server-side approach. This result has two important implications. First, not even the highly accurate GenerativeImage2Text is capable of being 100% “relevant”, according to our study participants. This is not unexpected, especially with no control over the input images, which can assume a very abstract form. It follows that the accuracy of the client-based approach is quite high, as it is only 10% worse from the best attainable by a sophisticated AI.

### What is the quality of AI-generated webpages?

This subsection investigates the quality of AI-generated webpages using user feedback collected on Prolific^22^. The feedback per image composing a webpage is collected first, and then the full webpages. Table 1 summarizes the user study. 10 user feedback for the two versions were collected (client-based and server-based) of the 409 images extracted from 60 websites randomly selected from the 200 previously crawled. Next, an additional 10 user feedbacks for two versions of each webpage are also collected, for a total of 9,400 user feedbacks. Prolific is asked to offer high-quality participants who are fluent in English so that they can understand the image descriptions. Among 940 study participants, the minimum *approval rate*, *i.e.,* the past rate of approval of their work, that was recorded, was 94%, with 80% of the participants having an approval rate of 99–100%.

**Fig. 4:**
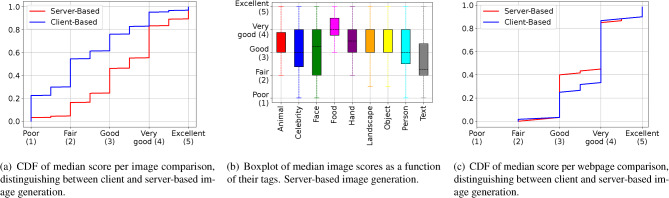
Results from the user study. *Client-based* refers to image generated on the client side, *i.e.,* only using contextual information such as alt text. *Server-based* refers to image generated on the server side, *i.e.,* assuming availability of both contextual information and original image.

The quality of AI-generated Web images is first investigated. Figure 4a shows the CDF of the median score (from 10 users) among images, distinguishing between client-based and server-based. The figure shows that, regardless of the approach, the majority of scores are *positive*, *i.e.,* “fair” or higher. Precisely, 95% of the median scores are better or equal to “fair” for server-based image generation; this number drops to 70% in the client-based approach. A reduction is expected given the more challenging conditions (lack of access to the original image) in which the client-based approach operates.

Next, the analysis digs deeper into the collected scores with respect to the *content* of the images. Each (original) image is manually tagged using the set of tags shown on the x-axis in Fig. 4b; limiting the analysis to a maximum of three tags per image, by considering the three predominant features. For example, the image in Fig. 2 is labelled as “object”. Note that the tags “person” and “face” are both observed which might seem redundant; however, these two tags are never used together, and their selection is based on whether a face is the main feature of an image. For example, a closeup of a person would be labelled as “face”, whereas a group of people working is labelled as “person”.

Figure 4b shows one boxplot per image label, where each boxplot contains the median scores of all the images tagged with such label. Only results for the server-based image generation scheme are shown since it shows a similar trend as the client-based approach. Focusing on the median, the figure shows that images containing “food”, “landscape”, and “object” achieve the best performance, with a median score of “very good” and rarely showing negative scores. Next, images labelled as “hand” or “animal” also perform well, with a median comprised between “good” and “very good”. It is worth noting that for all of the above categories, even the 25th percentile of the boxplot still achieves a score equal to or above “good”. When it comes to images related to humans (those labelled with “celebrity”, “person”, or “face”), it can be noticed that their median scores are still equal to or above “good”; however, their 25th percentile falls below that, even reaching a score of “fair” in the case of the “face” label. The only images whose median scores fall close to the “fair” score are those containing text. However, even in this category, the 25th percentile of the boxplot does not fall below the “fair” score. It is conjectured that these low scores are due to the fact that many of the texts generated by stable diffusion are either not completely readable or do not make sense due to the inability of stable diffusion to re-produce accurate text. The opposite is instead responsible for the higher scores, e.g., stable diffusion seems to be able to generate very convincing food images.

Next, the analysis investigates the quality of AI-generated webpages. Figure 4c shows the CDF of the median score (from 10 users) among webpages, distinguishing between the client-based and server-based approach. Compared to Fig. 4a, the overall score has improved, with no scores being “poor”, and only a few being “fair”. Indeed, 55-68% of the scores are either “very good” or “excellent”. The reason for the score increase compared to the previous image-to-image comparison is that webpages are more complicated, composed of a collection of text/images. While for the images study participants can focus on low-level details, such as potential hand deformity, this is less likely on a webpage where eyeballs tend to move quickly, potentially ignoring some images. The results of the AI-generated webpages show similar curves for both the client-based and the server-based, apart from the gap in the region between “good” and “very good”. In this region, about 10% of the images have scored higher in the case of client-based (“very good”) and lower in the case of server-based (“good”). It is conjectured that the quality difference of these images is so subtle, making it a very difficult and subjective decision on which of these two scores, “good” vs. “very good”, should these images be assigned.

To evaluate the reliability of the survey results, the Intraclass Correlation Coefficient (ICC)^54^ is computed. Specifically, the ICC(2,k) model is used given that: 1) the study participants are a sample of the target population (i.e., the population of the Prolific users); 2) each participant rates all presented questions; and 3) the main goal is to assess the mean reliability of the study participants. The ICC(2,k) results are shown in Table 2. As can be seen, the Intraclass correlation value was 0.756, indicating good inter-rater reliability^54^.

**Table 2:** Inter-rater reliability of our user study results.

	Intraclass Correlation	95% Confidence Interval		F Test with True Value 0			
		Lower Bound	Upper Bound	Value	df1	df2	P value
ICC(2,k)	0.756	0.57	0.89	4.8	19	209	$3.18 \times 10^{-9}$

**Fig. 5:**
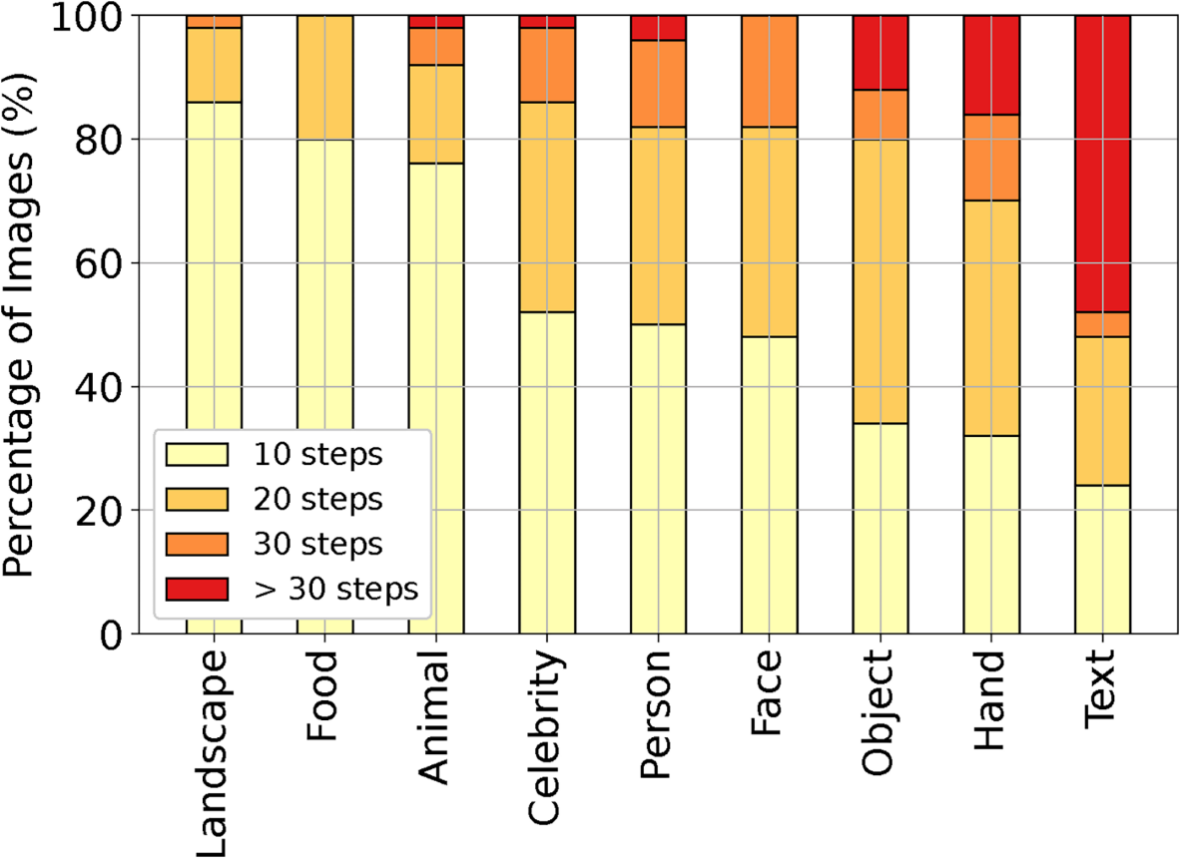
SDXL’s required inference step as a function of image tag to generate images with scores of Good and above.

To evaluate the number of inference steps required to generate “Good” images for each label, a manual annotation was conducted with five expert evaluators for a randomly selected subset of 50 images from each of the nine labels. The annotators first evaluated the 450 images (50 images x 9 labels) generated using an inference step size of 10. A majority vote per image was then taken, where at least three out of the five annotators gave that image at least a score of 3 (Good) or above. For the images that did not receive a majority vote of 3 or above, the annotators were asked to evaluate them again with inference step 20. If a majority vote was still not achieved, a final evaluation was conducted using inference step 30. Fig. 5 shows the results of the human annotation. For certain labels (Landscape, Food, and Animal), an inference step of 10 is enough to generate 80% of their images with a score quality of Good or above. For most of the remainder 20% of the images across these three labels, increasing the inference step to 20 is enough to achieve a score of Good or above. In contrast, for labels such as Celebrity, Person, Face, Object, and Hand, most images require an inference step of at least 20 to score Good or higher. Finally, more than 50% of the Text labeled images does not get a Good or above quality even with a 30 inference steps. In principle increasing the inference step beyond 30 could help in increasing the quality for certain labels. The above inference steps can be mapped directly into inference time (seconds) using Table 3, which shows inference times as a function of the number of inference steps and GPU type.

**Table 3:** SDXL’s inference time in seconds across multiple GPUs.

GPU	Inference Steps		
	10	20	30
v100	3.5 s	6.11 s	8.5 s
a40	3.33 s	4.98 s	6.83 s
a6000	2.66 s	4.67 s	6.5 s
a100	1.75 s	2.84 s	4.02 s

To evaluate the inter-rater reliability of the image quality annotations, the ICC is calculated per image label as can be seen in Table 4. The ICC values are derived using the ICC(3,k) model, which is appropriate for a fixed set of raters providing ratings on a common set of images^54^. The ICC values ranged from 0.681 to 0.799, indicating varying degrees of agreement across labels. The highest reliability is observed for Celebrity and Face images (ICC = 0.799, 95% CI [0.72, 0.86]), while the lowest reliability is observed for Animal images (ICC = 0.681, 95% CI [0.54, 0.79]). Notably, Landscape images have an ICC of 0.757 (95% CI [0.64, 0.84]), indicating good agreement among raters, while Food and Person images also exhibit strong reliability with ICC values of 0.779 and 0.724, respectively. Overall, all image label are characterized by statistically significant agreement, with p-values well below 0.05, further validating the consistency of the ratings across image types.

**Table 4:** ICC(3,k) Inter-rater reliability of our human annotation per image label.

	Intraclass Correlation	95% Confidence Interval		F Test with True Value 0			
		Lower Bound	Upper Bound	Value	df1	df2	P value
Landscape	0.757	0.64	0.84	4.1	57	228	$1.48 \times 10^{-14}$
Food	0.779	0.68	0.86	4.5	59	236	$5.35 \times 10^{-17}$
Animal	0.681	0.54	0.79	3.1	65	260	$5.47 \times 10^{-11}$
Celebrity	0.799	0.72	0.86	4.99	80	320	$2.68 \times 10^{-25}$
Person	0.724	0.62	0.81	3.6	83	332	$5.52 \times 10^{-17}$
Face	0.799	0.72	0.86	4.99	84	336	$1.72 \times 10^{-26}$
Object	0.788	0.71	0.85	4.73	92	368	$7.245\times 10^{-27}$
Hand	0.766	0.69	0.83	4.29	98	392	$5.24 \times 10^{-25}$
Text	0.709	0.62	0.79	3.4	113	452	$1.2 \times 10^{-20}$

### Can image generation run in the client?

The analysis is concluded by performing a reality check on running stable diffusion directly in the client. This technology offers several benefits, such as additional privacy and rectification of broken web pages, but comes at the cost of expensive hardware and/or potential webpage slowdowns. Table 3 have shown high inference times for SDXL even with superior GPUs such as the a100, where the inference time is $>=1.75$ second, these numbers suggests that the base model of SDXL might not be suitable to run in the client. However, recently a new version of SDXL has been proposed in literature, called SDXL Turbo^55^. This model is optimized to balance speed and quality, generating images in only 1-4 steps, which reduces the inference time significantly, making it the perfect candidate for this evaluation.

**Fig. 6:**
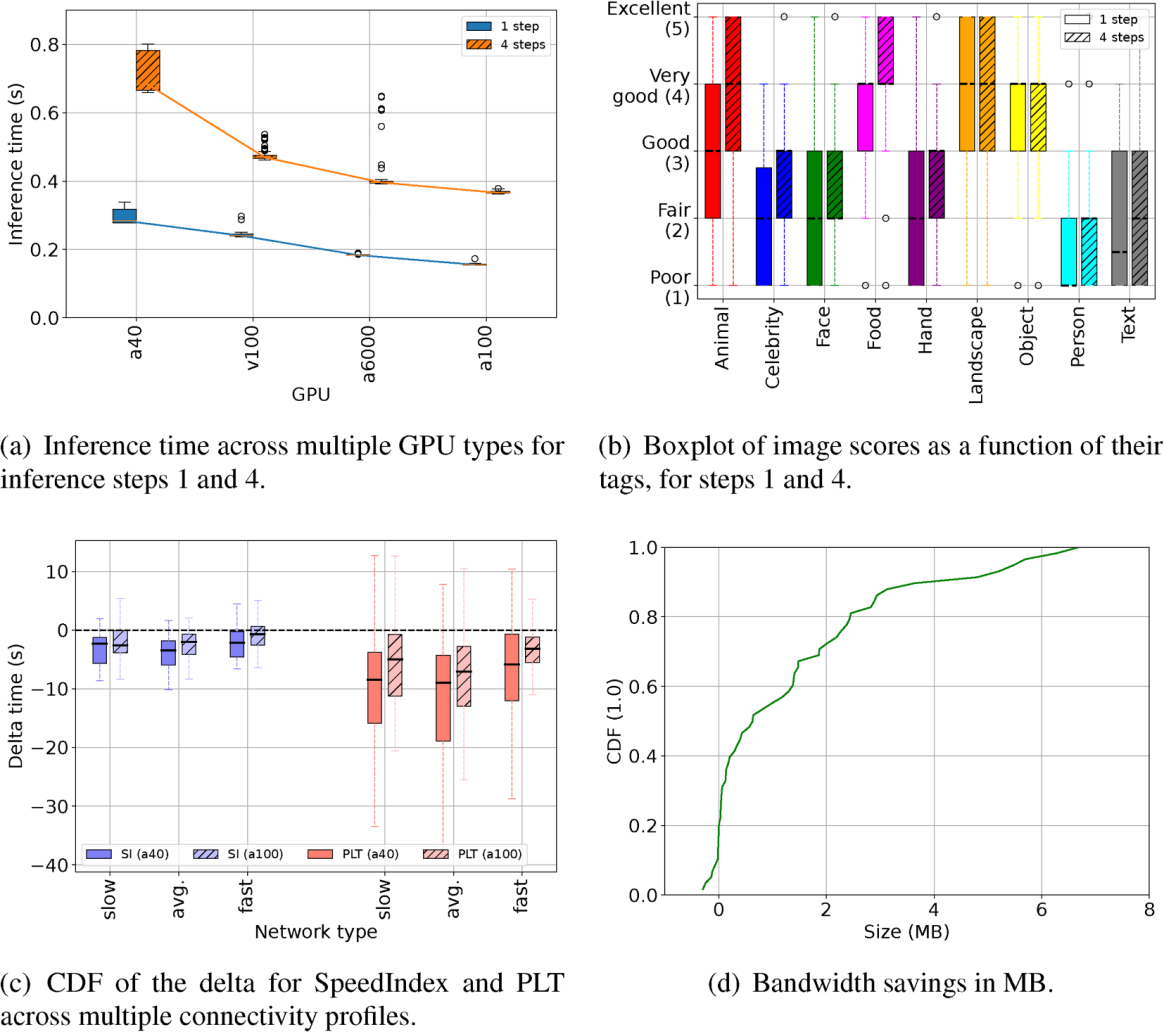
Reality check on running SDXL Turbo in the client for image generation.

First, we test SDXL Turbo’s inference time across various GPU types and step sizes. Figure 6a shows the boxplots of SDXL Trubo’s inference times, the results shows a significantly lower inference times of $<=0.7$ seconds even for the 4 steps when using the a40 GPU which is the slowest among all of the four investigated GPUs.

Second, we evaluated the quality of the images generated by SDXL Turbo across the nine image labels that we investigated earlier. To this end, we asked three human experts to score images on a scale of 1 (Poor) and 5 (Excellent), generated using SDXL Turbo using the same 50 randomly selected prompts for each image label, for both step sizes (1 and 4). As expected, given that SDXL Trubo was designed to balance speed and quality, Fig. 6b shows that certain image labels, such as Animal, Food, Landscape, and Object, received a median score of 3 (Good), whereas other labels related to Celebrity, Face, Hand, Person, and Text, had a lower median scores. The results also shows improved scores across all labels when the step size is increased to 4.

Next, we setup a set of experiments to assess whether SDXL Turbo can benefit the browsing experience when ran in the client. For these experiments, a powerful desktop machine similar to our stable diffusion server is assumed, *i.e.,* mounting powerful GPUs like the A40 or the A100, since a mobile phone’s hardware is far from capable of running such complex models. For example, as of June 2023, it took an iPhone-13 about 15.6 seconds to generate a single image using stable diffusion leveraging apple’s new core ML framework^56^ (this is viewed as a significant improvement compared to the 23 seconds generation time back in December 2022). For this reason, a typical speeds of home connections is emulated^57^, while ignoring slower speeds such as 3G. Network emulation is realized using TC (see Fig. 1) instructed via the following connectivity profiles: *slow* (symmetric 20 Mbps and 100 ms RTT), *average* (symmetric 50 Mbps and 50ms RTT), and *fast* (symmetric 100 Mbps and 20ms RTT). Chrome is used to load the previous 60 webpages (repeating each three times), both in their original form and considering AI-generated images. Textual prompts derived from the server-based scheme is used, although no difference is observed in the duration of image generation using the two approaches.

#### Web performance

Two popular Web performance metrics is utilized: SpeedIndex (SI) and Page Load Time (PLT)^49^. SI measures how quickly the visible content of a webpage is displayed during a page load. Conversely, PLT measures the total time it takes the browser to download and visualize the entire webpage, including content located below the fold. Figure 6c shows the CDF of the *delta* for each metric, derived as the difference between the metric computed for the original webpage and the metric computed for the webpage when images are AI-generated. It follows that a negative value indicates a slowdown, and a positive value indicates a speedup. Each delta value in the plot is the median out of three runs.

The figure shows that even with powerful GPUs, such as A40 and A100, generating images on the client using SDXL Turbo incur slight reductions compared to the actual image downloads when considering SI, a median of about 1 to 2 seconds slowdowns, with a100 (blue hatched boxplots) having relatively less slowdowns compared to a40 (blue solid boxplots). This happens because, on average, only 2-5 images are loaded above the fold, and the transport might need some time to converge to the available bandwidth, e.g., due to connection establishment and window increase. Furthermore, certain images positioned above the fold have substantial file sizes. However, when the PageLoadTime is considered, even on a very slow network, almost no webpage loads faster when images are locally generated. Whether this might change in the future is a question of how hardware and AI models would evolve, in contrast with network performance. Regardless, this result also suggests that locally generating images can be a viable solution for applications operating on a handful of images, such as privacy-preserving advertising and webpage repair – granted that powerful GPUs are adopted.

#### Resource usage

Figure 6d quantifies the bandwidth savings (MB) due to not transferring Web images, which are instead locally generated. Overall, the majority of tested webpages enjoy multiple MBs of savings, and even more than 4MB for the 10% heaviest webpages. These savings are marginal for devices on WiFi, the likely connectivity scenario for the assumed powerful devices. However, these savings can be significant for content providers. For example, let’s consider a popular newspaper with 1 million daily views; a median saving of 1 MB per view would account for daily savings of 1 TB.

## Discussion

### Copyright and ethical concerns

Our analysis shows that AI-generated images are highly realistic, making it difficult for users to distinguish between *real* and AI-generated content (see Fig. 5). While this is beneficial to the performance of a system adopting such technology, it can lead, for example, to the spread of misinformation if inappropriate images are generated. Similarly, AI models can inadvertently embed biases, which can also be dangerous for end-users^28^. It follows that websites or web browsers adopting AI-generated images need to disclose such behavior. For example, a browser or a website could add a watermark on images when AI-generated.

A second concern is copyright infringement. AI systems rely on large datasets, which may contain copyrighted material. Entities implementing this technology must ensure that their training data complies with copyright laws. Note that enforcing such copyright laws is a challenging task, as determining the originality and ownership of AI-generated content can be complex. This issue diverges from the focus of this paper and has been explored in recent research efforts such as Glaze^58^. Glaze enables artists to apply “style cloaks” to their artwork, introducing subtly imperceptible perturbations. When integrated into training datasets, these cloaks can deceive generative models attempting to replicate a particular artist’s style.

### Client or server deployment?

Our analysis has shown that, as of today, generative AI requires powerful GPUs to compete with the current Web model based on image caching and retrieval. As such, a server-based implementation is advisable, e.g., to speedup webpage creation, while still relying on classic image downloads. When considering few images and given the fast-paced performance improvement discussed in the Background and Related Work Section, generative AI can also be adopted directly in the client/browser, e.g., by leveraging the recent WebGPU API^59^. This is particularly true for applications which require client-side implementation, such as (fully) privacy-preserving advertising and webpage image repair.

Privacy-preserving advertising is a novel technology, for example, adopted by Brave^60^, where ad selection is performed in the browser, avoiding remote tracking mechanisms to build and auction user profiles^61^. Still, this mechanism requires a remote connection to fetch locally matched ads, which can cause some privacy leaks^62^. Client-side image (ad) generation allows for the avoidance of any remote communication offering full privacy. As discussed above for generic images, one potential side effect is a lack of accuracy with negative impact on the brand being advertised.

Webpage image repair is useful when a website misses some images due to local or third-party misconfigurations, such as broken links. In such cases, a client-side solution is recommended, as these issues typically stem from oversight by the website owner. Ideally, such disruptions should affect only isolated portions of the webpage, making the AI generation process feasible even on less powerful GPUs.

### User engagement

The deployment of generative AI for web images introduces a nuanced impact on user engagement, particularly when the quality of generated content does not match that of original page elements. Users often associate image quality with brand credibility and content relevance, making it crucial for generative AI applications to maintain standards that align with user expectations. Addressing these challenges effectively is essential for optimizing the user experience and maximizing the benefits of AI-driven image generation in web environments.

## Conclusion and future work

Over the past year, a significant increase in the adoption of generative Artificial Intelligence (AI) across a range of fields is observed, including medicine and media production. Similarly, web developers and browser vendors are turning to generative AI for various applications, such as multimedia asset creation, webpage acceleration, and privacy enhancements. This paper explored the role of generative AI in the specific context of web image generation and consumption. To accomplish this, WebDiffusion is introduced, a tool that facilitates real-time experiments using unaltered web browsers, thus enabling an in-depth assessment of the quality and effectiveness of AI-generated images within target webpages. Moreover, by seamlessly integrating with crowdsourcing platforms such as Prolific, WebDiffusion offers a valuable avenue for collecting feedback and insights from a diverse global audience. Our results indicate that generative AI can already create relevant and high-quality web images without the need for manual prompts from web designers, relying on contextual information found within webpages. Conversely, in-browser image generation remains a challenging task, as it requires highly capable GPUs like the A40 and A100 to partially match traditional image downloads. Nevertheless, this approach holds promise for small-scale image generation tasks, such as repairing broken webpages or serving privacy-preserving media.

This paper opens several interesting avenues for future work. First, stable diffusion often produces artefacts when generating images of faces and hands even with a large number of iterations (> 100, increasing the image generation time to above 15 seconds), especially when there are more than one subject in the produced image. While stable diffusion is constantly improving in this respect, one could consider extending WebDiffusion to other text-to-image generation models. For example, the GFPGAN model is a popular method that addresses the issue of distorted faces. This model helps restore faces in samples and improves the overall realistic quality of the generated image^63^.

Next, this paper currently only focuses on image generation. While images are a fundamental component of webpages and account for a large fraction of the page size, there are other elements, such as HTML, CSS, and JavaScript, that can be (partially) automatically generated. Such generation is, however, more challenging as, differently from images, it can impact Web compatibility, *i.e.,* break the functioning of webpages. Nevertheless, with the constant improvement of generative AI, it is possible to envision how these tools can be used in a broader sense for the Web, and this work thus plan to extend WebDiffusion to support them.

## Data Availability

The datasets generated during and/or analysed during the current study are available from the corresponding author upon request.
